# A systematic review and meta-analysis of homocysteine concentrations in chronic obstructive pulmonary disease

**DOI:** 10.1007/s10238-022-00833-0

**Published:** 2022-05-05

**Authors:** Angelo Zinellu, Elisabetta Zinellu, Maria Carmina Pau, Alessandro G. Fois, Sabrina Mellino, Barbara Piras, Valentina Scano, Sara S. Fois, Arduino A. Mangoni, Ciriaco Carru, Pietro Pirina

**Affiliations:** 1 Department of Biomedical Sciences, University of Sassari, 07100 Sassari, Italy; 2 Clinical and Interventional Pulmonology, University Hospital Sassari (AOU), 07100 Sassari, Italy; 3 Department of Medical, Surgical and Experimental Sciences, University of Sassari, v.le San Pietro 43, 07100 Sassari, Italy; 4 Department of Clinical Pharmacology, College of Medicine and Public Health, Flinders University and Flinders Medical Centre, Bedford Park, SA 5042 Australia

**Keywords:** COPD, Homocysteine, Comorbidities, Cardiovascular risk

## Abstract

Patients with chronic obstructive pulmonary disease (COPD) often suffer from other conditions, such as cardiovascular disease, that further increase the risk of adverse outcomes in this group. Serum homocysteine concentrations are positively associated with cardiovascular risk and have also been reported to be increased in COPD. This meta-analysis investigated the association between homocysteine concentrations and COPD. A systematic search of publications in the electronic databases PubMed, Web of Science, Scopus, and Google Scholar, from inception to September 2021, was conducted using the following terms: “Homocysteine” or “Hcy” and “Chronic Obstructive Pulmonary Disease” or “COPD”. Weighted mean differences (WMDs) were calculated to evaluate differences in homocysteine concentrations between COPD patients and non-COPD subjects. Risk of bias and certainty of evidence were assessed using the Joanna Briggs Institute Critical Appraisal Checklist and GRADE, respectively. Nine studies in 432 COPD patients (mean age 65 years, 65% males) and 311 controls (mean age 65 years, 56% males) were identified. Pooled results showed that serum homocysteine concentrations were significantly higher in patients with COPD (WMD = 2.91 µmol/L, 95% CI 2.00–3.82 µmol/L; *p* < 0.001; high certainty of evidence). No publication bias was observed. Our results support the hypothesis that increased homocysteine concentrations are significantly associated with COPD and may account, at least in part, for the increased cardiovascular risk in these patients.

## Introduction

Chronic obstructive pulmonary disease (COPD) is the third leading cause of death worldwide [1]. In 2017, there were ~ 270 million cases of COPD and ~ 18 million were newly reported globally [2]. COPD is an inflammatory condition characterized by persistent respiratory symptoms and airflow obstruction due to both small airways disease and parenchymal destruction [3]. The airflow limitation is progressive and not completely reversible and appears to be the result of a chronic inflammatory state that worsens with the progression of the disease [4, 5]. Cigarette smoking and other irritants stimulate macrophages and airway epithelial cells to release mediators of inflammation that are responsible for the recruitment of inflammatory cells. In this context, an important role is also played by oxidative stress, a condition where reactive oxygen species (ROS) overcome the antioxidant defence systems [6, 7]. Inflammatory cells release ROS that activate proinflammatory molecules, leading to the recruitment of further inflammatory cells. Moreover, ROS activate NF-κB which, in turn, activates several inflammatory genes and impairs the function of antiproteases, such as *α*1-antitrypsin, accelerating the breakdown of elastin in the lung parenchyma [4, 6]. The prognosis of patients with COPD is often complicated by the presence of comorbidities [8, 9]. For example, co-existing cardiovascular disease leads to high rates of morbidity and risk of hospitalisation and mortality in patients with COPD [10]. It is widely established that elevated serum or plasma concentrations of the sulfur-containing amino acid homocysteine, an intermediate in the metabolism of the essential amino acid methionine, increase cardiovascular risk [11–13]. Homocysteine causes endothelial dysfunction through the induction of inflammation and oxidative stress, both involved in the pathogenesis of COPD, and the inhibition of nitric oxide synthesis [14–16]. Homocysteine concentrations can increase mainly as the result of specific genetic defects of the enzymes responsible for homocysteine metabolism as well as deficiencies of cofactors involved in this pathway such as vitamins B6, B12 and folic acid. Such deficiencies are secondary to reduced intake or absorption and use of specific medications [17, 18]. Recent studies have shown that homocysteine concentrations are also increased in COPD patients, particularly in those with severe disease and rapid progression [19, 20]. Therefore, to critically appraise the available evidence on the link between homocysteine and COPD, we conducted a systematic review and meta-analysis of homocysteine concentrations in serum or plasma of COPD patients and in subjects without COPD.

## Materials and methods

### Search strategy, eligibility criteria, and study selection

A systematic search of publications in the electronic databases PubMed, Web of Science, Scopus and Google Scholar, from inception to September 2021, was conducted using the combination of the following terms: “Homocysteine” or “Hcy” and “Chronic Obstructive Pulmonary Disease” or “COPD”. Two investigators screened the abstracts to establish relevance and, if so, the full text was reviewed according to the following inclusion criteria: (i) assessment of serum homocysteine concentrations; (ii) comparison of subjects with and without COPD (case–control design); (iii) adult participants; (iv) English language; and (v) full text available. The references of the retrieved articles were also searched for additional studies. Any discordance between the two investigators was resolved by a third investigator. The risk of bias was evaluated using the Joanna Briggs Institute (JBI) Critical Appraisal Checklist for analytical cross-sectional studies, with scores ≥ 5, 4 and < 4 indicating low, moderate and high risk, respectively [21]. The certainty of evidence was assessed using the Grades of Recommendation, Assessment, Development and Evaluation (GRADE) Working Group system. GRADE considers the study design (randomized vs. observational), the risk of bias (JBI checklist), the presence of unexplained heterogeneity, the indirectness of the evidence, the imprecision of the results (sample size, 95% confidence interval width and threshold crossing), the effect size (WMD) and a high probability of publication bias [22–24]. The assessment of the potential biological and clinical significance of the WMD was based on prospective studies reporting that a 10% increase in homocysteine concentrations is associated with an adjusted hazard ratio for cardiovascular events between 1.87 and 1.97 [25]. For a baseline homocysteine concentration of 15 µmol/L, the WMD was therefore considered high (>  − 1.5 µmol/L, 10% reduction), moderate (between − 0.75 and − 1.5 µmol/L, 5–10% reduction) and low (<  − 0.75 µmol/L, < 5% reduction), respectively. We fully complied with the Preferred Reporting Items for Systematic reviews and Meta-Analyses (PRISMA) 2020 statement on the reporting of systematic reviews and meta-analyses [26].

### Statistical analysis

Weighted mean differences (WMDs) and 95% confidence intervals (95% CIs) were calculated to generate forest plots of continuous data and to evaluate differences in homocysteine concentrations between COPD patients vs. non-COPD subjects (a p-value of less than 0.05 was considered statistically significant). When necessary, the mean and standard deviation values were extrapolated from median and interquartile range values, as previously reported by Wan et al. [27] or from graphs using the Graph Data Extractor software. Heterogeneity of WMD values across studies was assessed using the Q-statistic (significance level set at a p-value of less than 0.10). The I^2^ statistic, a quantitative measure of inconsistency across studies, was also calculated with values < 25% indicating no heterogeneity, between 25 and 50% moderate heterogeneity, between 50 and 75% large heterogeneity and > 75% extreme heterogeneity) [28, 29]. Statistical heterogeneity was defined as an *I*^2^ statistic value ≥ 50% [29]. In analyses in which heterogeneity was high, a random-effects model was used. Sensitivity analysis was conducted to investigate the influence of individual studies on the overall risk estimate by excluding them sequentially [30]. To evaluate the presence of publication bias, the associations between study size and magnitude of effect were analyzed using the Begg’s adjusted rank correlation test and the Egger’s regression asymmetry test, with a significance level set at a p-value of less than 0.05 [31, 32]. Statistical analyses were performed using Stata 14 (STATA Corp., College Station, TX, USA). The study protocol was registered in the International Prospective Register of Systematic Reviews (PROSPERO registration number: CRD42021282004).

## Results

### Systematic research and study characteristics

**Fig. 1:**
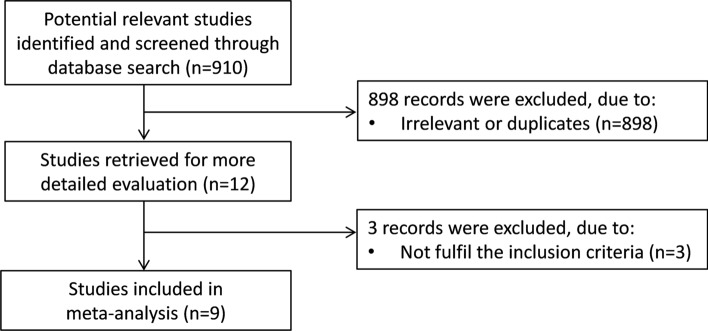
Flow chart of study selection

A flow chart describing the screening process is presented in Fig. 1. We initially identified 910 studies. A total of 898 were excluded after the first screening because they were either duplicates or irrelevant. After a full text review of the remaining 12 articles, three were further excluded because they did not meet the inclusion criteria. Therefore, nine studies in 432 COPD patients (mean age 65 years, 65% males) and 311 controls (mean age 65 years, 56% males), published between 2001 and 2020, were included in the final analysis (Table 1) [19, 20, 33–39]. In eight studies, the diagnosis of COPD was made according to the Global Obstructive Lung Disease (GOLD) guidelines [19, 20, 34–39]. No information regarding the tool used for COPD diagnosis was provided in the remaining study [33].

**Table 1:** Study characteristics

First author year, country	Non-COPD subjects				COPD patients				
	*n*	Age Mean	Gender (M/F)	Homocysteine Mean ± SD (µmol/L)	*n*	Age Mean	Gender (M/F)	Homocysteine Mean ± SD (µmol/L)	Diagnosis
Andersson A et al. 2001, Sweden	29	64	14/15	14.1 ± 4.9	19	68	8/11	17.9 ± 6.7	NR
Kai S et al. 2006, Japan	23	63	NR	9.8 ± 3.0	24	71	NR	12.6 ± 2.9	GOLD
Seemungal TAR et al. 2007, England	25	65	16/9	8.1 ± 2.2	29	69	23/6	10.7 ± 4.5	GOLD
Abdallah GM et al. 2009, Egypt	20	NR	12/8	7.6 ± 1.3	24	NR	18/6	9.4 ± 1.3	GOLD
Fimognari FL et al. 2009, Italy	29	71	21/8	11.9 ± 2.9	42	71	36/6	14.8 ± 4.7	GOLD
Kahn NA et al. 2016, India	30	58	13/17	15.2 ± 15.7	50	52	43/7	27.4 ± 23.9	GOLD
Moayyedkazemi A et al. 2018, Iran	51	66	29/22	18.2 ± 9.5	40	67	22/18	19.5 ± 9.5	GOLD
Wei B et al. 2020, China	50	58	28/22	7.5 ± 2.7	150	62	90/60	11.7 ± 2.7	GOLD
Zinellu A et al. 2020, Italy	54	73	40/14	13.0 ± 3.7	54	73	40/14	15.5 ± 3.8	GOLD

GOLD, Global initiative for chronic obstructive lung disease; NR, Not reported; COPD, chronic obstructive pulmonary disease; M, male; F, female; SD, standard deviation

### Risk of bias

The risk of bias was considered low in all studies (Table 2).

**Table 2:** The Joanna Briggs Institute critical appraisal checklist

Study	Were the criteria for inclusion in the sample clearly defined?	Were the study subjects and the setting described in detail?	Was the exposure measured in a valid and reliable way?	Were objective, standard criteria used for measurement of the condition?	Were confounding factors identified?	Were strategies to deal with confounding factors stated?	Were the outcomes measured in a valid and reliable way?	Was appropriate statistical analysis used?	Risk of bias
Andersson A	Yes	Yes	Yes	Yes	No	No	Yes	No	Low
Kai S	Yes	Yes	Yes	Yes	No	No	Yes	No	Low
Seemungal TAR	Yes	Yes	Yes	Yes	Yes	Yes	Yes	Yes	Low
Abdallah GM	Yes	Yes	Yes	Yes	No	No	Yes	No	Low
Fimognari FL	Yes	Yes	Yes	Yes	Yes	Yes	Yes	Yes	Low
Kahn NA	Yes	Yes	Yes	Yes	No	No	Yes	No	Low
Moayyedkazemi A	Yes	Yes	Yes	Yes	No	No	Yes	No	Low
Wei B	Yes	Yes	Yes	Yes	No	No	Yes	No	Low
Zinellu A	Yes	Yes	Yes	Yes	Yes	Yes	Yes	Yes	Low

### Results of individual studies and syntheses

**Fig. 2:**
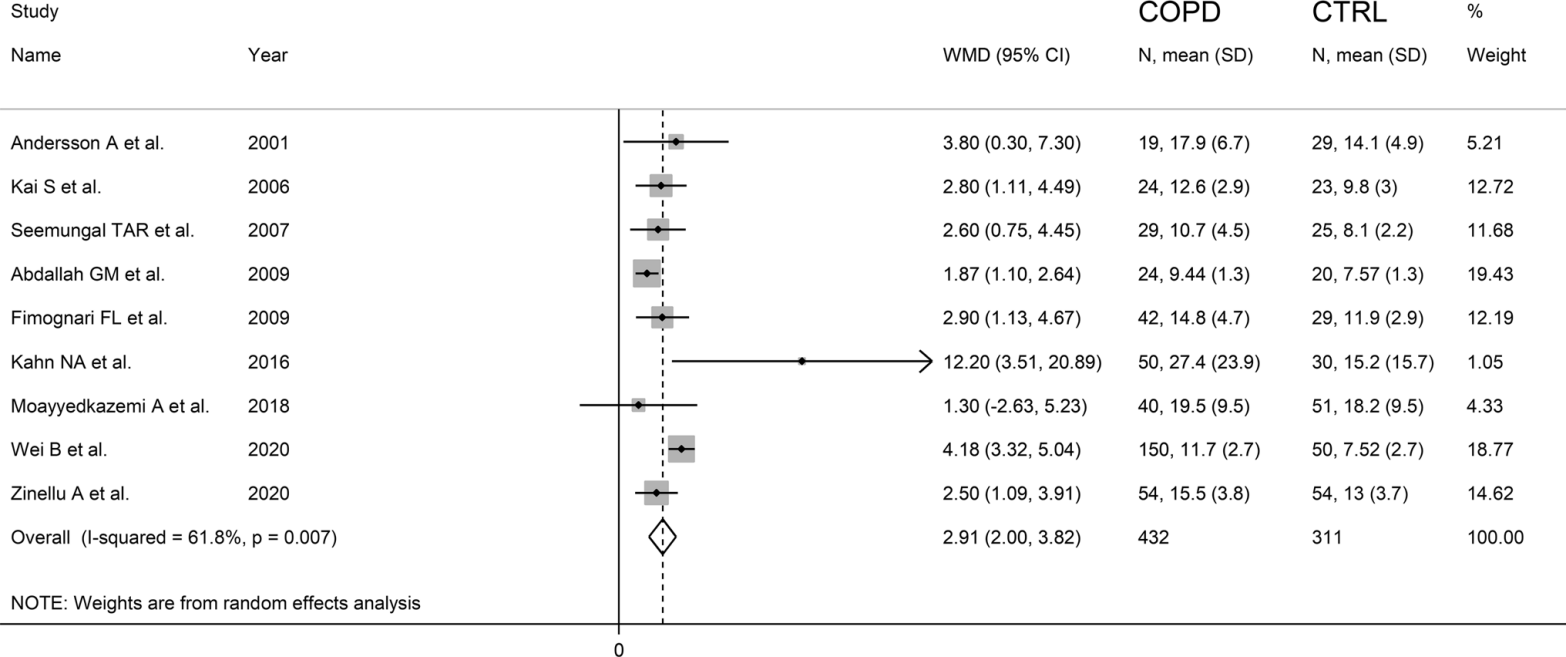
Forest plot of serum homocysteine concentrations in COPD patients and non-COPD subjects

**Fig. 3:**
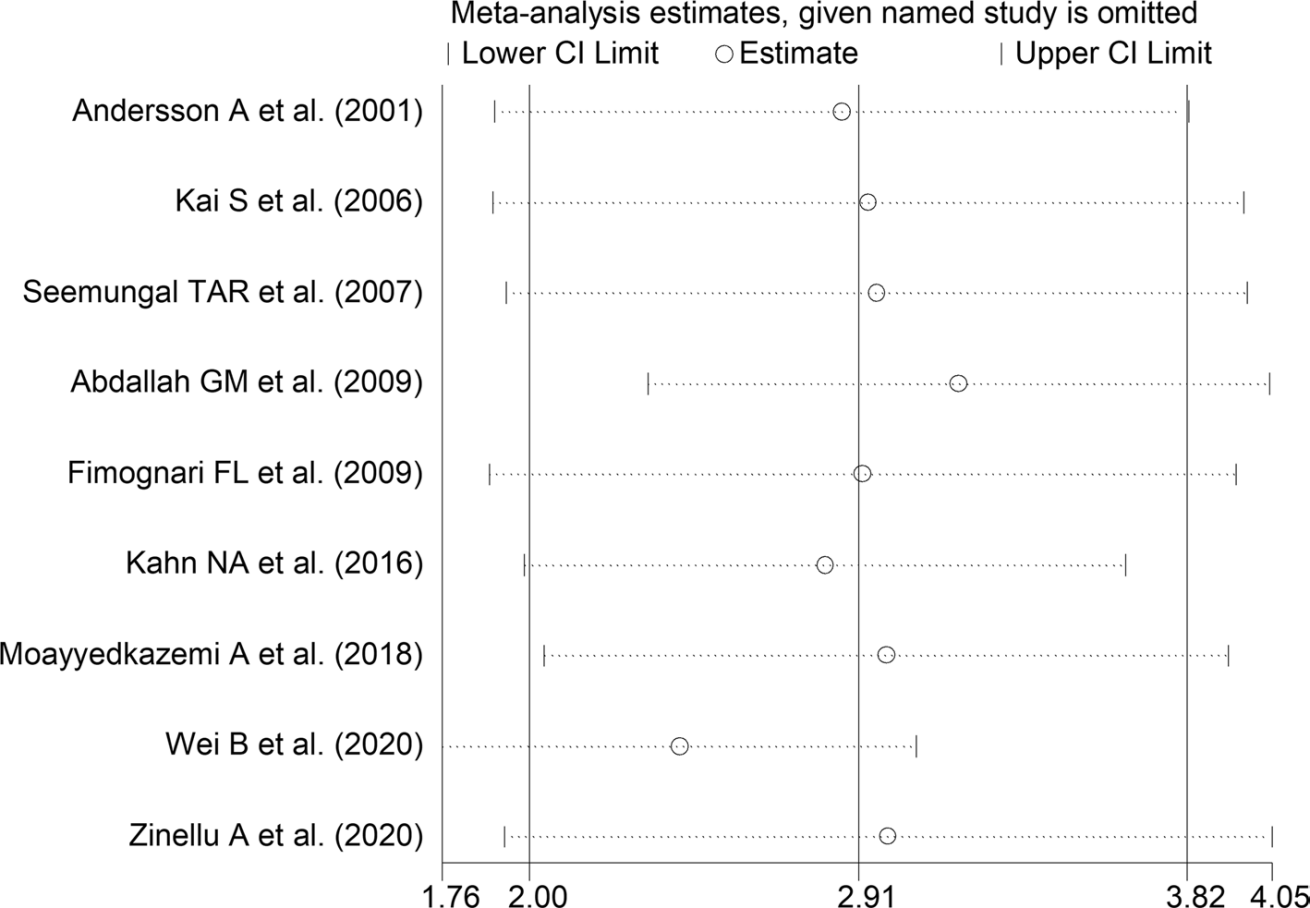
Sensitivity analysis of the association between serum homocysteine and COPD. The influence of individual studies on the overall weighted mean difference (WMD) is shown. The middle vertical axis indicates the overall WMD, and the two vertical axes indicate the 95% confidence intervals (CIs). The hollow circles represent the pooled WMD when the remaining study is omitted from the meta-analysis. The two ends of each broken line represent the 95% CIs

**Fig. 4:**
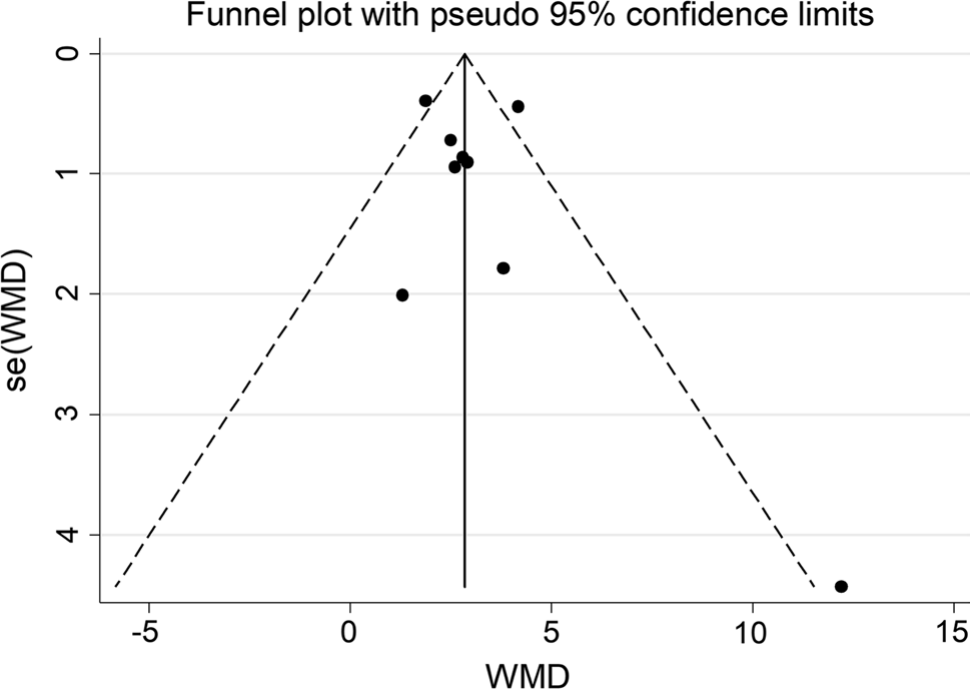
Funnel plot of studies investigating serum homocysteine in COPD patients and non-COPD subjects

**Fig. 5:**
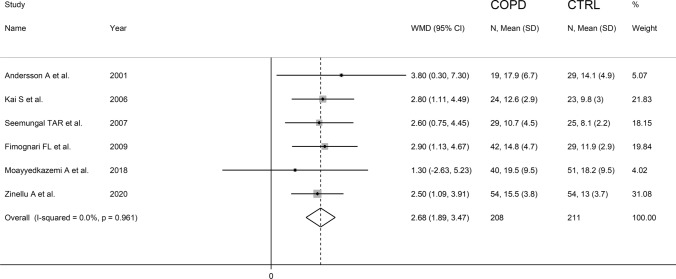
Forest plot of studies examining serum homocysteine in COPD patients and non-COPD subjects after removing three outliers

The forest plot for serum homocysteine concentrations in COPD patients and non-COPD subjects is shown in Fig. 2. In all studies, COPD patients had higher homocysteine concentrations compared to non-COPD subjects with a significant difference reported in all but one [38]. A large heterogeneity between studies was observed; thus, random-effects models were used. Overall, pooled results showed that homocysteine concentrations were significantly higher in patients with COPD (WMD = 2.91 µmol/L, 95% CI 2.00–3.82 µmol/L, *p* < 0.001). Sensitivity analysis showed that the corresponding pooled WMD values were not substantially altered when each study was sequentially removed (Fig. 3). Funnel plot analysis showed three outliers (Fig. 4). After removing these studies [34, 36, 38], the WMD remained significant (WMD = 2.68 µmol/L, 95% CI 1.89–3.87 µmol/L, *p* < 0.001) but with a virtually absent heterogeneity (Fig. 5).

### Publication bias

No publication bias was observed either with the Begg’s (*p* = 0.47) or the Egger’s (*p* = 0.55) test.

### Certainty of evidence

The initial level of certainty for homocysteine WMD values was considered low because of the cross-sectional nature of the studies (rating 2, ⊕  ⊕  ⊝ ⊝). After considering the low risk of bias in all studies (no rating change required), a generally extreme heterogeneity that was virtually eliminated after removing a subgroup of three studies (no rating change required), the lack of indirectness (no rating change required), the relatively low imprecision (relatively narrow confidence intervals without threshold crossing, upgrade one level), the relatively large effect size (upgrade one level) and the absence of publication bias (no rating change required), the overall level of certainty was considered high (rating 4, ⊕  ⊕  ⊕ ⊕).

## Discussion

This is an updated systematic review and meta-analysis that reports for the first time a significant association between homocysteine concentrations and the presence of COPD. Mild-to-moderate hyperhomocysteinemia is relatively common in the general population, especially in older adults, and is mainly associated with deficiencies in group B vitamins, such as vitamin B12, vitamin B6 and folic acid which serve as cofactors in homocysteine metabolism [17, 18]. As COPD is also characterized by complex nutritional deficiencies [40], it is plausible that the presence of a poor folic acid status may lead to elevated homocysteine concentrations in these patients. This hypothesis, however, requires further research as a study failed to provide evidence that hyperhomocysteinemia in COPD patients represented a reliable marker of folate and vitamin B12 deficiencies [41]. Nevertheless, another study showed a reduction of homocysteine concentrations in COPD patients after folate supplementation [38]. Moreover, several epidemiological studies have reported significant and positive associations between homocysteine concentrations and cardiovascular disease, an important comorbidity that also shares some risk factors with COPD, such as cigarette smoking and increasing age which are also associated with high homocysteine concentrations [17]. The results of our study further support the presence of an association between homocysteine and COPD. A similar meta-analysis, published in 2019 by Chaudhary et al. [42], included only four studies on 145 COPD patients and 107 non-COPD subjects and failed to report significant between-group differences in homocysteine concentrations. Our study included a greater number of articles, nine, and subjects, 432 COPD patients and 311 non-COPD subjects. The overall result showed the presence of significantly higher concentrations of homocysteine in COPD patients compared to non-COPD subjects. The high heterogeneity observed may be explained by several unreported factors, such as differences in sample handling, storage conditions and analytical procedures as well as geographical location, ethnicity, smoking status and presence of comorbidities. Specifically, three studies contributed to the reported heterogeneity. Their removal virtually eliminated the heterogeneity while maintaining significant between-group differences in homocysteine. Additional limitations include the lack of subgroup analysis to identify specific associations between effect size and other clinical or analytical characteristics, as this information was not provided. Despite these limitations, this meta-analysis reports an association between homocysteine and COPD presence with a high level of certainty and without publication bias. Moreover, in sensitivity analysis, the pooled WMD values were not substantially altered when individual studies were sequentially removed. Some of the studies also reported that homocysteine concentration was positively related to COPD severity [19, 20]. However, their limited number prevented the conduct of additional pooled analyses. Our findings support the hypothesis that increased homocysteine concentrations are associated with COPD and that such association may at least partially account for the increased cardiovascular risk in these patients. In recent studies, plasma concentrations of homocysteine > 10 µmol/L have been shown to be associated with the development of cardiovascular disease [43]. Shiao et al. reported that for each 5 μmol/L increase in homocysteine, the risk of mortality increased by 32%, and the risk of heart disease increased by 52% [44]. Another meta‐analysis showed that a 3 µmol/L decrease in serum homocysteine concentrations was associated with a 16% reduction in coronary heart disease and that a 5 µmol/L increase was associated with a 1.6–1.8 fold risk of coronary heart disease [45]. Several experimental and human studies have shown that homocysteine may disrupt endothelial and vascular homeostasis by causing increased vascular smooth muscle cell proliferation, oxidative damage, and alterations of coagulation [46].

In conclusion, our updated systematic review and meta-analysis has shown that COPD patients have significantly higher homocysteine concentrations when compared to non-COPD controls. This might at least partially explain the common occurrence of atherosclerotic cardiovascular disease in COPD patients given the well-known deleterious effects of homocysteine on endothelial function and vascular homeostasis. However, further research is warranted to investigate the presence of additional factors mediating the association between homocysteine and COPD and whether interventions targeting this highly reactive amino acid improve respiratory and cardiovascular outcomes in this group.
